# Integration of Serum Metabolomics into Clinical Assessment to Improve Outcome Prediction of Metastatic Soft Tissue Sarcoma Patients Treated with Trabectedin

**DOI:** 10.3390/cancers12071983

**Published:** 2020-07-21

**Authors:** Gianmaria Miolo, Emanuela Di Gregorio, Asia Saorin, Davide Lombardi, Simona Scalone, Angela Buonadonna, Agostino Steffan, Giuseppe Corona

**Affiliations:** 1 Medical Oncology and Cancer Prevention Unit, Centro di Riferimento Oncologico di Aviano (CRO), IRCCS, 33081 Aviano, Italy; gmiolo@cro.it (G.M.); dlombardi@cro.it (D.L.); sscalone@cro.it (S.S.); abuonadonna@cro.it (A.B.); 2 Immunopathology and Cancer Biomarkers Unit, Centro di Riferimento Oncologico di Aviano (CRO), IRCCS, 33081 Aviano, Italy; emanuela.digregorio@cro.it (E.D.G.); asia.saorin@unive.it (A.S.); asteffan@cro.it (A.S.)

**Keywords:** metabolomics, soft tissue sarcomas, prognosis, overall survival, biomarkers, citrulline

## Abstract

Soft tissue sarcomas (STS) are a group of rare and heterogeneous cancers with few diagnostic or prognostic biomarkers. This metabolomics study aimed to identify new serum prognostic biomarkers to improve the prediction of overall survival in patients with metastatic STS. The study enrolled 24 patients treated with the same trabectedin regimen. The baseline serum metabolomics profile, targeted to 68 metabolites encompassing amino acids and bile acids pathways, was quantified by liquid chromatography-tandem mass spectrometry. Correlations between individual metabolomics profiles and overall survival were examined and a risk model to predict survival was built by Cox multivariate regression. The median overall survival of the studied patients was 13.0 months (95% CI, 5.6–23.5). Among all the metabolites investigated, only citrulline and histidine correlated significantly with overall survival. The best Cox risk prediction model obtained integrating metabolomics and clinical data, included citrulline, hemoglobin and patients’ performance status score. It allowed to distinguish patients into a high-risk group with a low median overall survival of 2.1 months and a low- to moderate-risk group with a median overall survival of 19.1 months (*p* < 0.0001). The results of this metabolomics translation study indicate that citrulline, an amino acid belonging to the arginine metabolism, represents an important metabolic signature that may contribute to explain the high inter-patients overall survival variability of STS patients. The risk prediction model based on baseline serum citrulline, hemoglobin and performance status may represent a new prognostic tool for the early classification of patients with metastatic STS, according to their overall survival expectancy.

## 1. Introduction

Soft tissue sarcomas (STS) are a rare, heterogeneous group of tumors of mesenchymal origin that account for about 1% of all adult malignancies [1]. More than 100 histological subtypes, from different tissues of origin and with different clinical behaviors, have been identified [2]. Leiomyosarcomas and liposarcomas, together called L-sarcomas, are the most common subgroup, representing about one third of all adult cases [3,4,5].

Surgery is the standard primary treatment for localized, resectable STS [6,7], while first-line chemotherapy with doxorubicin, alone or in combination with ifosfamide, is the treatment of choice for metastatic STS [8]. Trabectedin has been approved in Europe and the United States for metastatic STS patients after failure of anthracycline-based chemotherapy or for patients who are unsuited to receive these agents [5]. Trabectedin has been found to be most effective against L-sarcomas and translocation-related sarcoma subtypes, but this efficacy does not always translate into longer overall survival [3,4,9,10,11,12]. Retrospective studies found that second-line treatment with trabectedin was associated with a median overall survival of 12.2 months; however, there was high inter-patient variability in survival with only 8% of the patients alive at 48 months [13,14].

To date, only a few prognostic factors have been identified for STS treated with trabectedin [15,16,17,18]. Low histological grade, absence of liver metastasis, young age and good performance status seem to predict a better outcome, while low circulating levels of sodium, hemoglobin and albumin and high absolute neutrophil count (ANC) have been associated with a worse outcome [15,17]. However, the prognostic power of these clinical factors is limited and better pre-therapeutic biomarkers to guide clinical decision-making are needed.

New approaches for serum biomarker research are provided by metabolomics, an emerging omics field focused on the analysis of the entire set of metabolites present in biological samples [19]. The main outcome of metabolomics studies is the metabolite profile of the biological system under investigation. Such a profile describes the biochemical events occurring in an organism and it reflects the complex interactions among age, sex, gene transcription, protein expression, physio-pathological conditions, including gut microbiome activity and environment effects [20,21]. These features make metabolomics an important tool to assess the patients’ phenotypes and it has already been widely used to identify diagnostic cancer signatures and prognostic biomarkers [22,23,24,25]. However, only a few metabolomics studies have been reported for sarcoma and they mainly regard in vitro investigations [26,27]; those performed in a clinical setting were focused on specific sarcoma subgroups [28,29,30] and limited only to gemcitabine treatment [31]. In this prospective study, we used a metabolomics approach to search for new serum prognostic markers for overall survival in patients with metastatic STS treated with trabectedin. In particular, we determined the serum levels of 68 targeted metabolites at baseline and integrated metabolomics into clinical data to develop a risk model for overall survival after trabectedin treatment.

## 2. Results

### 2.1. Study Population

This metabolomics study enrolled 24 patients with metastatic STS scheduled for treatment with trabectedin. The study group included equal numbers of men and women with a median age of 59 years (Table 1). Leiomyosarcoma was the most prevalent histotype (*n* = 8, 33.3%). A grade 2 tumor had been diagnosed in 8 cases, while the remaining cases had a G3 tumor. Performance status, according to the Eastern Cooperative Oncology Group (ECOG) score, was 0 in 13 cases (54.2%) and 1 in 11 cases (45.8%), indicating overall good wellbeing. This, and normal renal, hepatic and bone marrow functions, were prerequisites for trabectedin treatment. All patients had already had chemotherapy with anthracyclines or gemcitabine and here were scheduled for trabectedin as a second-line (75%) or third-line (25%) treatment.

At data analysis, 20 patients had died and 4 were still alive. The median overall survival was 13.0 months (95% CI, 5.6–23.5) with wide inter-patient variability ranging from 0.8 to 47 months. Survival in patients with L-sarcomas (median, 23.5 months; 95% CI, 4.1–33.2) was significantly longer than in patients with other sarcomas (11.3 months; 95 % CI, 1.7–25.8; *p* = 0.046 log-rank test) (Figure S1a). Patients with a performance status (PS) score of 0 had significantly longer survival than those with PS = 1 (23.5 months [95% CI, 5.6–25.8] vs. 7.6 months [95% CI, 2.1–33.2]; *p* = 0.026) (Figure S1b). Finally, patients with hemoglobin <12 g/dL had a shorter median survival time of 4.1 months (95% CI, 0.8–24.2) than that of patients with hemoglobin ≥12 g/dL (21.8 months; 95% CI, 7.6–33.2; *p* = 0.001) (Figure S1f). No association between overall survival and other investigated variables (tumor grade, age, ANC, sodium, albumin, lactate dehydrogenase) was found.

### 2.2. Metabolite Profiles and Association with Overall Survival

The metabolic phenotype of each patient, before trabectedin treatment, was investigated by targeted profiling of 53 amino acids and their derivatives and 15 bile acids in serum. This analysis generated serum molar concentrations of each metabolite for each patient (Table S1). Of the 68 investigated metabolites, 54 (79%) were quantified while 14 amino acids (21%) were absent or below the level of detection of the assay.

Metabolites correlation visualized in hierarchical clustering heat map, revealed only one cluster of metabolites whose levels changed together in the patients (Figure S2). This group comprised the primary and secondary bile acids conjugated with taurine and glycine. With the exclusion of this specific metabolic pattern, the serum concentrations of most of the investigated metabolites are independent and, thus, can be used to describe each patient’s phenotype.

**Figure 1:**
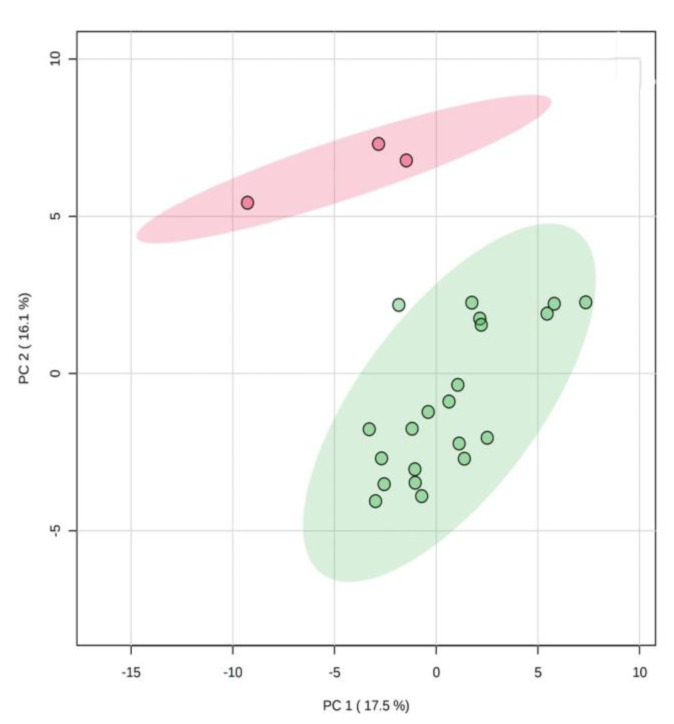
Principal component analysis (PCA) scores plot derived from serum metabolomics profiles of 24 patients with metastatic soft tissue sarcomas (STS). The patients clustered into two distinct groups, one with only 3 cases (red) and the other with the remaining 21 cases (green).

To determine if these metabolomics profiles could be broadly used to distinguish patients into subgroups, we used multivariate principal component analysis (PCA). This analysis identified two clusters, one with only three patients, and the other with the remaining 21 patients, whose serum metabolomics profiles were significantly different (Figure 1). The metabolites that most contributed to the spatial separation were prevalently amino acids, while no bile acids were involved. In particular, the three profiles of the small cluster had lower levels of threonine, methionine, arginine, phenylalanine, histidine and citrulline. Interestingly, all three patients had low trabectedin responsiveness STS (fibrosarcoma, *n* = 1; undifferentiated pleomorphic sarcoma, *n* = 2), with G3 tumor grade and they experienced a poor clinical outcome, with overall survival <4.7 months.

### 2.3. Identification of Metabolomics Signatures of Overall Survival

In order to find metabolites whose serum levels were associated with overall survival, the quantitative metabolomics data were screened by univariate Cox proportional hazards regression (Table 2). Two amino acids resulted to be significantly associated with overall survival (FDR < 0.05): the proteinogenic amino acid citrulline and the essential amino acid histidine.

**Figure 2:**
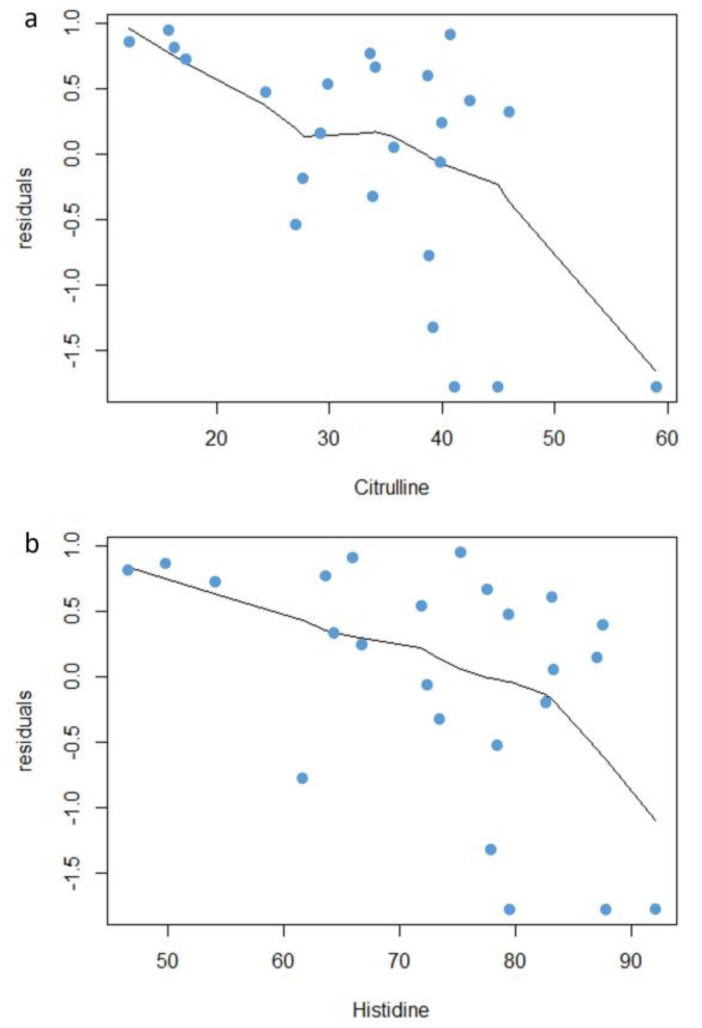
Scatter plot of the Martingale residuals for the metabolites citrulline (**a**) and histidine (**b**). Blue points represent individual patients and the curve was fitted by the locally weighted scatterplot smoothing (LOWESS) smoother.

To check the residual association between the overall survival and these two covariates, the Martingale residuals from the Cox proportional hazard regression were plotted against the levels of citrulline (Figure 2a) and histidine (Figure 2b). Patients with largest residuals above the zero had increased risk for death, and those below a decreased risk for death compared with the expected risk from Cox regression model. Long survival and short survival patients were distinguished according to a cutoff value that in Martingale residuals plot corresponds to the inflection point of the curve. This cutoff for citrulline was clearly recognizable at about 30 µM and for histidine at about 75 µM, suggesting that these metabolites could have a role in the overall survival estimate.

The differences in baseline serum levels of citrulline and histidine were also investigated in relation to the clinical and tumor characteristics of patients. Only for citrulline was found a significant association with the tumor grade. Serum citrulline levels were significantly lower in patients with a G3 tumor grade as compared to those with a G2 tumor (mean ± SD, µM, 30.7 ± 10.7 and 39.8 ± 9.6, *p* = 0.03). No other clinical features, including previous chemotherapy regimens, resulted significantly associated with the baseline level of citrulline.

### 2.4. Risk Prediction Model Development

A risk prediction model was developed integrating the metabolomics data with clinical characteristics such as tumor grading, histotype, PS and blood test parameters selected by univariate Cox proportional hazards regression (*p* < 0.05).

Subsequent backward multivariate Cox regression with these variables identified three as having a significant, independent impact on survival: citrulline, hemoglobin and PS (Table 3). These variables were used to build the risk prediction model, described by the following equation:

(1) $$\ln\left(\frac{H\left(t\right)}{H_{0}\left(t\right)}\right)=-0.084\cdot \left[\mathrm{Citrulline}\right]-0.388\cdot [\mathrm{Hb}]+1.117\cdot \left[\mathrm{PS}\right]$$

In the model, H(t)/H0(t) and its natural log represent the hazard ratio and the risk score, respectively. For variables with a negative coefficient (citrulline and hemoglobin), as these values increase, overall survival is predicted to increase. Instead, for PS, which has a positive coefficient, as this value increases overall survival is predicted to decrease. The Cox regression model showed a Harrell’s C-index of 0.80, demonstrating the goodness of fit of the model. Citrulline had a hazard ratio of 0.92; however, it resulted important to predict overall survival. Its contribution was investigated removing it from the risk prediction model and re-evaluating the overall model fit. The Harrell’s C-index resulted significantly decreased when citrulline was excluded (from 0.80 to 0.75, *p* = 0.02), underlining its value as prognostic factor. Risk scores for the 24 patients ranged from a low of −10.81 to −3.36, with a median of −7.11 (Figure S3). Six patients had a risk score greater than the 75th percentile value (from −6.19 to −3.36) and thus formed the high-risk (H-Risk) group; the other patients formed the low- to moderate-risk (LM-Risk) group.

**Figure 3:**
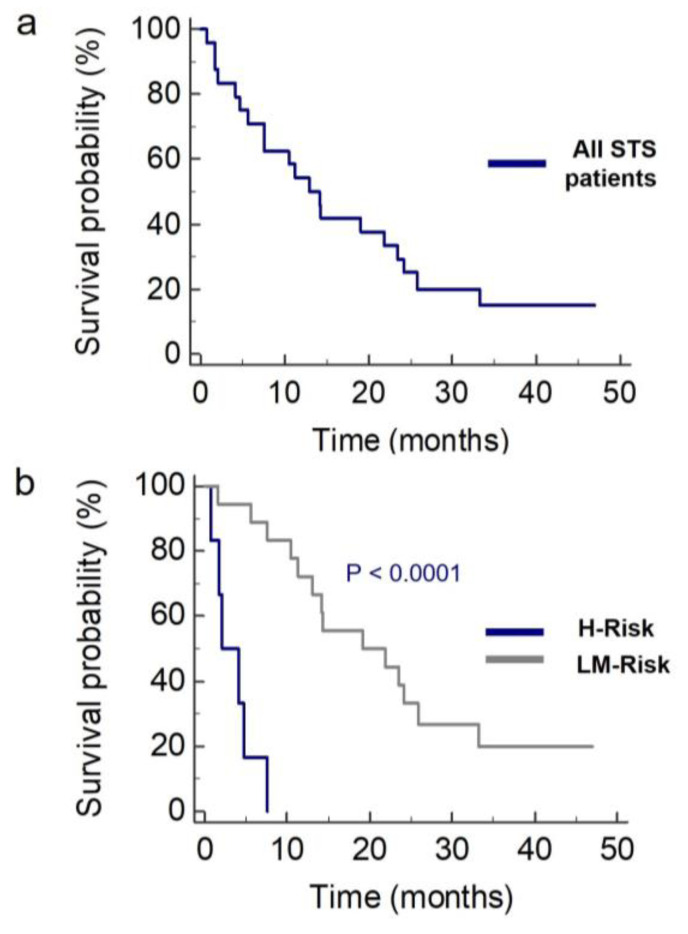
Kaplan–Meier survival analysis for overall survival in all patients with metastatic STS (**a**), and in the high-risk (H-R, *n* = 6) and low- to medium-risk (LM-R, *n* = 18) group identified according to their risk score (**b**). Median survival was 2.1 months (95 % CI, 0.8–7.6) and 19.1 months (95% CI, 11.3–25.8), respectively.

Kaplan-Meier survival analysis was used to test the proportional hazard assumption of the Cox model for the two risk groups (Figure 3). The median overall survival for the H-Risk group was 2.1 months (95% CI, 0.8–7.6), while for the LM-Risk group it was 19.1 months (95% CI, 11.3–25.8), confirming the association between overall survival and risk score calculated with the model (*p* < 0.0001, log-rank test). The diagnostic power of the two circulating components in the model, in distinguishing the two risk groups, was calculated by receiving operator curve analysis (Figure S4). For citrulline, this analysis gave an area under the curve (AUC) of 0.93 (95% CI, 0.74–0.99), a sensitivity of 100 % and a specificity of 77.8% with a cut-off value of 33.7 µM. For hemoglobin, the AUC was 0.87 (95% CI, 0.67–0.97), sensitivity was 100%, and specificity was 72.2% with a cut-off value of 12.4 g/dL.

### 2.5. Arginine Metabolism by Risk Group

**Figure 4:**
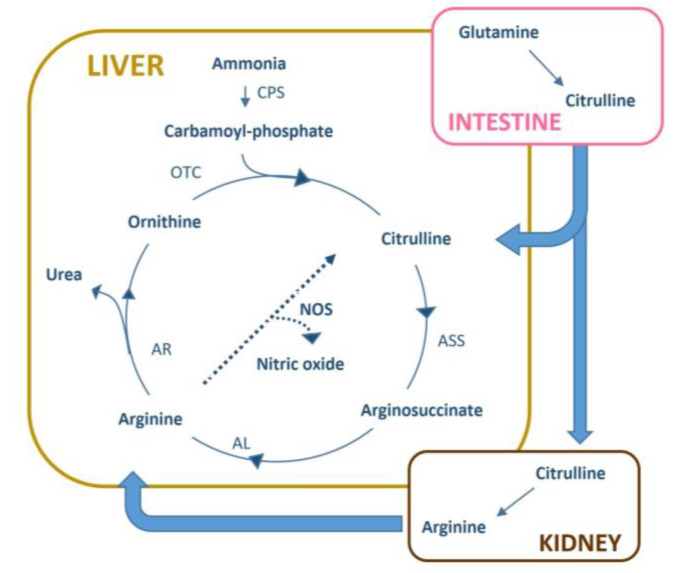
Metabolic pathways involving citrulline and arginine. Abbreviation: CPS, carbamoyl phosphate synthetase; ASS, argininosuccinate synthase; AL, argininosuccinate lyase; AR, arginase; OTC, ornithine transcarbamoylase.

Citrulline belongs to the arginine metabolic pathway (Figure 4). It is produced by enterocytes from glutamine and released into the blood where is taken up by kidney for the synthesis of arginine or goes to the liver where it participates in the transformation of ammonia to urea [32].

**Figure 5:**
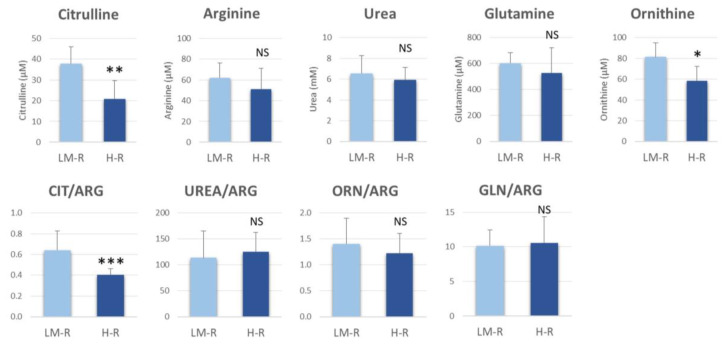
Serum concentrations of metabolites involved in arginine metabolic pathways and their ratios by risk group. Values are mean and SD. *** *p* < 0.001; ** *p* < 0.01; * *p* < 0.05, Student’s *t* test. LM-R, low- to medium-risk group; H-R, high-risk group; CIT, citrulline; ARG, arginine; ORN, ornithine; GLN, glutamine; NS, not significant.

The differences between H-Risk and LM-Risk groups in serum levels of citrulline, arginine, urea, glutamine and ornithine were investigated (Figure 5). Besides citrulline, a significant difference was also found for ornithine (*p* = 0.02, Student’s *t* test). These metabolites were 0.43 and 0.77 fold lower in the H-Risk than LM-Risk group, respectively. To better manage the inter-patient variability, the ratios of the metabolites to arginine were calculated. Only the citrulline:arginine (CIT/ARG) ratio was significantly (*p* < 0.0001) different between groups. Urea levels did not differ significantly between the investigated groups, suggesting that ammonia excretion by the urea cycle is not affected. Moreover, the low citrulline levels that characterized the H-Risk group were not associated with a lack of the precursor glutamine.

## 3. Discussion

Clinical outcome prediction in metastatic STS patients is challenging and there is a strong need to improve the current knowledge about the principal factors that contribute to determine the high inter-patient variability to treatments. The metabolomics profile, with its intrinsic characteristics able to integrate host and tumor biochemical as well as environmental information, represents a very attractive approach to study the individual patient’s phenotype associated with the high inter-patient overall survival variability.

In this study of STS patients treated with trabectedin, the median overall survival was 13.0 months (95% CI, 5.6–23.5) superimposable to those reported in previous studies [5,13,33]. The patients had a wide variability in survival that was partially explained by tumor histology, PS and baseline hemoglobin, confirming previously reports [15,16,34,35]. Conversely, in our study survival did not associate with ANC, albumin or sodium, which previously had been reported to be negative prognostic factors of trabectedin outcome [15,17]. The difference between the results of this investigation and those of previous reports may be due to the better PS of patients in our series.

The novelty of the current study is the use of the metabolomics profile to explain the variability in overall survival and to find new STS prognostic biomarkers. Serum amino acids and their derivatives along with primary and secondary bile acids were targeted for metabolomics profiling. Amino acids are involved in almost all cellular biochemical pathways, so they provide an overall “snapshot” of a patient’s metabolic status [36,37,38,39], while bile acids and their conjugated derivatives reflect liver and gut microbiome activity [40]. This targeted metabolomics approach, although limited to two classes of serum metabolites, allowed us to capture specific patients’ phenotypes. Indeed, when patients’ metabolic profiles were investigated by multivariate PCA analysis a cluster of patients with low overall survival was identified. Correlation analysis between serum metabolite concentrations and overall survival revealed positive correlations for the amino acids citrulline and histidine, indicating that high serum levels of both amino acids are associated with better prognosis. However, when metabolomics and clinical features were integrated in Cox regression model, citrulline emerged as the only metabolic biomarker able to predict overall survival. In addition, the clinical variables hemoglobin and PS were also independent predictors of survival included in the model. These results, besides confirming hemoglobin as a prognostic factor of trabectedin treatment in STS patients [15], underline the emerging role of serum citrulline in determining patient’s survival. The integrated clinical-metabolomics risk model allowed us to distinguish the patients into two groups: a H-Risk group with low overall survival (median, 2.1 months) and a LM-Risk group with longer overall survival (median, 19.1 months, *p* < 0.0001). The role of citrulline was further underscored by its high diagnostic power evaluated by receiving operator characteristic (ROC) analysis, which better distinguished the H-Risk and LM-Risk groups than did hemoglobin. In our study population, the H-risk patients could be identified by the following clinical metabolic cut-off values: citrulline ≤ 33.7 µM, hemoglobin ≤ 12.4 g/dL and PS ≥ 1.

The observed citrulline shortage in H-Risk patients may be the result of tumor-patient metabolic interplay. Indeed, in cancer patients, the metabolic reprogramming that sustains cancer cell proliferation, survival and metastasis not only involves cancer cells but also affects the whole host organism [24]. A tumor is a high energy-demanding tissue that requires, besides glucose, other carbon intermediates, which may result in deficiencies of certain metabolites in the blood.

Citrulline metabolism occurs in several organs, including the liver where it participates in the urea cycle for eliminating ammonia, the kidney where it is a precursor for the de novo synthesis of arginine and the intestine where it is synthesized from glutamine [32,41]. Due to this localized biosynthesis, citrulline is a biomarker of intestinal function, since its serum decrease is associated with a reduced enterocyte mass consequent to bowel inflammation [42,43] or damage from chemotherapy [44]. The lower serum citrulline in the H-Risk group may reflect altered intestinal function not associated with a shortage of glutamine, given that the level of this latter did not differ significantly between groups. H-Risk patients do not seem to have dysbiosis that alters intestinal function and citrulline biosynthesis, since the bile acid profiles were not significantly different between the two groups of patients. Serum urea levels were similar in the two groups, suggesting the absence of alterations in liver efficacy for the metabolic elimination of ammonia through the urea cycle. This evidence is supported by the similarities between groups in the urea:arginine and ornithine:arginine metabolic ratios. Thus, the lower circulating levels of citrulline and ornithine in the H-risk group may be not related to a urea cycle alteration but more likely to their high consumption. The increased utilization of ornithine in H-risk patients may be due to a higher polyamine synthesis. However, this hypothesis could not be verified since the serum levels of the polyamines spermine, spermidine and putrescine were below the limits of detection of this metabolomics analysis. Alternatively, the high citrulline consumption in the H-risk group could be linked to a homeostasis mechanism aimed to maintain a steady level of serum arginine. A high citrulline-to-arginine conversion by transamination in H-Risk patients is supported by the citrulline:arginine ratio, which was significantly lower than in the LM-Risk group.

Although studies of cancer cell metabolism have focused on aerobic glycolysis, several other metabolic pathways are emerging as important for tumor development. In STS, altered expression of arginine-metabolizing enzymes has been reported [45]. In particular, the low expression of argininosuccinate synthetase-1 (ASS1), which is involved in arginine synthesis from citrulline, was found as the principal cause of arginine auxotrophism that characterized almost all STS histotypes. The high systemic conversion of citrulline to arginine in the H-Risk group may reflect an aggressive STS form with a high metabolic demand of arginine to maintain its growth. This may be the cause of the observed shortage of citrulline associated with low overall survival of the H-Risk group. Interestingly, low serum citrulline levels were observed in patients with G3 tumor grade, supporting the hypothesis that a decrease of this metabolite may be associated with a more aggressive disease. However, not all high-grade tumors patients showed a shortage in citrulline likely because among the G3 sarcomas there could be a great heterogeneity in the auxotrophy for arginine that may determine a lack of a strong correlation between the tumor grade and serum citrulline shortage.

The link between such metabolic feature and the efficacy of trabectedin appears to be difficult to explain since arginine metabolism is not directly involved in the antitumor activity of the drug. Most likely, the imbalance in citrulline-arginine metabolism seems to delineate a specific metabolic phenotype that is the result of the complex host-tumor interplay and does not influence trabectedin activity but rather reflects the individual ability to contrast the effect of the disease. In this context, the prognostic value of citrulline could be independent from the kind of chemotherapy and even from the type of cancer. In support of this hypothesis, there are previous studies that reported reduced levels of citrulline in patients with high-grade serous ovarian cancer [46] and among patients with non-small cell lung cancer undergoing immunotherapy treatment [47], where low baseline levels of serum citrulline were associated with short overall survival. These reports support that citrulline shortage may be a negative prognostic marker for cancer overall and not limited only to STS or to specific chemotherapy treatment.

The low sample size and the lack of an independent cohort of patients are the major limitations of the current study that prevent a formal validation of the risk prediction model. Further investigations with a larger patient population are needed to verify the role of citrulline as a new prognostic biomarker in patients with tumors different from STS. Furthermore, to prove that citrulline may be a predictive factor independent from the chemotherapy, additional investigations on STS patients undergoing different treatments are needed.

## 4. Materials and Methods

### 4.1. Chemicals

Acetonitrile and methanol (LC-MS grade) were purchased from Carlo Erba Reagents (Milan, Italy). Formic acid, ammonium acetate and ammonium formate were obtained from Merck Life Science (Milan, Italy). Ultrapure water was generated by a Milli-Q Plus system (Millipore, Billerica, MA, USA). The Bile Acids LC-MS/MS kit, consisting of five calibrators, three levels of quality controls and labeled internal standards, was acquired from Biocrates Life Sciences (Innsbruck, Austria). Analytical reference standards and labeled internal standards for amino acid quantification were purchased from Toronto Research Chemicals (North York, ON, Canada).

### 4.2. Patients, Clinical Data and Blood Sampling

The study included 24 patients with metastatic STS who were scheduled for treatment with trabectedin (as second- or third-line therapy) from 2016 to 2019 at the Centro di Riferimento Oncologico di Aviano. To qualify for trabectedin treatment, patients had to have adequate renal, hepatic and bone marrow function and an ECOG performance status score ≤1. Trabectedin was administered intravenously during 24 h at the dose of 1.5 mg/m^2^ body surface area every 21 days for 6 cycles or until disease progression. The study protocol was approved by the Ethics Committee of Centro di Riferimento Oncologico di Aviano (project identification code: 2015.004CE, 09/04/2015, NCT04394728). The study was conducted in accordance with the Declaration of Helsinki and all patients gave written informed consent.

Prior to the first trabectedin infusion, venous blood (5 mL) was collected in glass tubes and was allowed to clot for 30 min at room temperature. Samples were then centrifuged at 4 °C for 10 min at 1900 g (Thermo Scientific Heraeus Megafuge 16R centrifuge), and the upper serum phase was immediately stored at −80 °C until analysis.

For each patient, we collected from clinical records the following baseline data: age at blood sampling, sex, body mass index (BMI), tumor histotype and grade, ECOG performance status and hematological parameters.

### 4.3. LC-MS/MS Metabolomics Analysis

#### 4.3.1. Bile Acid Analyses

Serum was profiled for 15 bile acids, including two primary bile acids (cholic acid, chenodeoxycholic acid), three secondary bile acids (deoxycholic, lithocholic and ursodeoxycholic acids) and 10 taurine- or glycine-conjugated derivatives (Table S1). Profiling was done by high-performance liquid chromatography using an Agilent 1290 Infinity II binary pump, coupled with an Ultivo triple quadrupole mass spectrometer (Agilent Technologies, Santa Clara, CA, USA) equipped with an electrospray ionization source (ESI). Briefly, 20 μL of serum was mixed with 5 μL of labeled internal standards mixture (Biocrates Life Sciences). Serum protein was precipitated by adding 40 μL of acetonitrile, vortexing vigorously and centrifuging at 20,800 g for 10 min at 4 °C. The supernatant was diluted 1:1.5 with ultrapure water and transferred to an auto-sampler glass vial; 10 μL was injected into a C18 reverse phase column kept at 50 °C. Mobile phase A consisted of 10 mM ammonium acetate 0.02% formic acid in water and mobile phase B was 10 mM ammonium acetate 0.02% formic acid in 65% acetonitrile and 35% methanol. Chromatographic separation was performed by gradients elution: (a) 35% to 40% B in 0.7 min; (b) 40% to 45% in 2.3 min; (c) 45% to 55% B in 0.2 min; (d) 55% to 65% B in 2.3 min; and (e) 65% to 100% B in 1 min. A 2 min washing step with 100% B and 3 min of equilibration to the initial condition (35% B) preceded the next sample injection.

The MS parameters were optimized in order to have the highest signals for the ESI source along with the MRM transitions selected for the negative ions. The best ionization source conditions for the Agilent ESI Jet Stream source were: capillary, 3000 V; nozzle voltage, 0 V; gas temperature, 200 °C; gas flow, 12 L/min; nebulizer, 40 psi; sheath gas temperature, 200 °C; and sheath gas flow, 10 L/min. MS/MS signals were integrated using MassHunter Quantitative Analysis software (Agilent) and quantified using a calibration curve with low-, medium- and high-quality plasma controls. Measures of intra-assay and inter-assay variability for each bile acid investigated were <15%.

#### 4.3.2. Amino Acid Analyses

Serum was also profiled for 53 free amino acids and derivatives (Table S1) according to the method of Prinsen et al. [48] with a few modifications. Briefly, 10 μL of serum was mixed with 20 μL of a solution of internal standards including 34 deuterated or ^13^C- or ^15^N-labeled amino acids in ultrapure water. Then, 150 μL of acetonitrile–0.1% formic acid (75:25, v/v) was added to precipitate serum proteins. The tubes were vortexed vigorously and centrifuged at 20,800 g for 15 min at 4 °C. Then, 6 μL of the supernatant was injected into the LC-MS/MS system. Amino acids were separated by hydrophilic interaction liquid chromatography on an XBridge Amide column 3 × 100 mm, particle size, 3.5 μM (Waters, Milford, MA, USA). The column was kept at 10 °C and equilibrated with 20% mobile phase A (20 mM ammonium formate pH 3) and 80% mobile phase B (acetonitrile containing 10% water and 20 mM ammonium formate pH 3) delivered at a flow rate of 0.2 mL/min. The gradient started at 80% B and reached 65% B at 10 min; this was followed by an isocratic step of 1 min at 20% B before returning to the initial condition of 80% B at 12.1 min. An equilibration time of 5 min with a flow of 0.7 mL/min was allowed before the next sample was injected. Each batch of analyses included a calibration curve and two levels of quality controls for the quantification. The ESI Jet Stream source operated in positive polarity in MRM mode with the following parameters: capillary, 2000 V; nozzle voltage, 0 V; gas temperature, 150 °C; gas flow, 10 L/min; nebulizer, 40 psi; sheath gas temperature, 400 °C; and sheath gas flow, 10 L/min. Measures of intra-assay and inter-assay variability for each amino acid investigated were <15%.

### 4.4. Risk Prediction Model Building and Testing

To build a risk prediction model, univariate Cox proportional hazard regression was first used to identify metabolites and clinical parameters significantly associated with overall survival that was calculated from the date of starting trabectedin chemotherapy to death or last follow-up.

Then, the significant variables with *p* < 0.01 were selected and further screened by multivariate Cox proportional hazards regression to build the model. Covariates were included in the Cox model by backward selection to test the independent significance of different variables. Specifically, all covariates with *p* < 0.05 were entered into the model and non-significant variables were removed sequentially. A prognostic model based on significant metabolites was constructed to calculate a risk score for each patient using the following equation [49]:

(2) $$\ln\left(\frac{H\left(t\right)}{H_{0}\left(t\right)}\right)=b_{1}X_{1}+b_{2}X_{2}+\ldots +b_{n}X_{n}$$

where *H(t)/H0(t)* is the hazard ratio, its natural log is the risk score and *b_n_* are the regression coefficients from multivariate Cox regression. The significance of the model was assessed using the Wald test and its goodness-of-fit was measured with Harrell’s concordance index (C-index). Risk scores were used to stratify patients into quartiles, with a high-risk group comprising the fourth quartile and a low- to medium-risk group comprising the first three quartiles. The proportional hazard assumption of the Cox regression model was verified using Kaplan-Meier curves. The log-rank test was used to compare survival curves between the groups defined by the risk score. The diagnostic power of individual variables in the model, to distinguish the two risk groups, was tested by receiving operator characteristic (ROC) curves.

### 4.5. Statistical Analyses

Univariate Kaplan–Meier survival analyses were performed to identify associations between overall survival and: tumor type (L-sarcomas vs. others), performance status (0 vs. 1), tumor grade (G2 vs. G3), age (<65 vs. ≥65 years), ANC (<7.5 vs. ≥7.5∙10^9^ cells/L), hemoglobin (<12 vs. ≥12 g/dL), albumin (<3.5 vs. ≥3.5 g/L) and serum lactate dehydrogenase (<320 vs. ≥320 U/L). The log-rank test was used to compare survival curves.

Unsupervised multivariate principal component analysis (PCA) of serum metabolite data was used to determine if the patients clustered into distinct groups. Hierarchical clustering of metabolites in a heat map, according to Pearson’s correlation coefficient for every possible combination, was performed to identify patterns of interaction among the metabolites. Univariate Cox regression analysis was used to screen significant metabolites associated with overall survival. Martingale residuals were calculated from Cox regression model for the most significant covariates (FDR < 0.05) to assess their relationship with the overall survival and to establish a possible cutoff for short and long survivors. *p*-values adjustment for FDR associated with multiple hypothesis testing was performed by Benjamini–Hochberg approach.

Student’s *t* test was used to compare metabolite concentrations and their ratios between subgroups of patients. Statistical tests were done using MedCalc statistical software, version 19.2.1, R version 4.0.0 and MetaboAnalyst 4.0. A *p* < 0.05 was considered statistically significant unless otherwise specified.

## 5. Conclusions

This investigation supports the use of serum metabolomics to search for new prognostic biomarkers predictive of the outcome of metastatic STS patients treated with trabectedin. The most important finding of this translational metabolomics study is that citrulline emerged as a potential biomarker of clinical outcome. The integration of the metabolomics data with clinical assessment led to the development of a risk model based on citrulline, hemoglobin and PS, which predicts overall survival and should allow the early identification of patients that may receive the best benefit from trabectedin treatment.

## Figures and Tables

**Table 1:** Clinical characteristics of 24 patients with metastatic soft tissue sarcoma.

Variable	Value
Sex, *n* (%)	
Female	12 (50.0)
Male	12 (50.0)
Age (years), median, range	59 (37–72)
Age, *n* (%)	
<65 years	14 (58.3)
≥65 years	10 (41.7)
BMI (kg/m^2^), median (range)	26.4 (16.6–35.3)
Tumor subtype, *n* (%)	
L-sarcomas ^a^	9 (37.5)
Other sarcomas ^b^	15 (62.5)
Grade, *n* (%)	
G2	8 (33.3)
G3	16 (66.7)
Performance status (ECOG score), *n* (%)	
0	13 (54.2)
1	11 (45.8)
Trabectedin therapy, *n* (%)	
2nd line	18 (75)
3rd line	6 (25)
Absolute neutrophil count, 10^9^ cells/L, mean (SD)	5.3 (3.2)
Sodium, mmol/L, mean (SD)	141.2 (1.8)
Albumin, g/dL, mean (SD)	3.8 (0.4)
Lactate dehydrogenase, U/L, mean (SD)	382.2 (212.8)
Hemoglobin, g/dL, mean (SD)	12.5 (1.8)
Red blood cells, 10^6^ cells/µL, mean (SD)	4.2 (0.6)
Monocytes, 10^3^ cells/µL, mean (SD)	0.6 (0.7)

BMI, body mass index; ECOG, Eastern Cooperative Oncology Group. ^a^ Leiomyosarcomas (*n* = 8) and liposarcoma (*n* = 1). ^b^ Other sarcomas include: malignant peripheral nerve sheath tumor (*n* = 3), fibrosarcoma (*n* = 2), undifferentiated pleomorphic sarcoma (*n* = 2), chondrosarcoma (*n* = 2), synovial sarcoma (*n* = 2), not otherwise specified sarcoma (*n* = 2), endometrial stromal sarcoma (*n* = 1), and desmoplastic small-round-cell tumor (*n* = 1).

**Table 2:** Univariate Cox regression analysis of the baseline metabolites.

Covariate	*p*	FDR *	Exp(b)	95% CI
Citrulline	0.001	0.018	0.907	0.86–0.96
Histidine	0.006	0.035	0.942	0.90–0.98
Cystathionine	0.011	0.053	11.343	1.75–73.43
TCA	0.023	0.070	41.431	1.67–1.03·10^3^

* *p*-value corrected by Benjamini and Hochberg false discovery rate (FDR) procedure. Exp(b), relative risk; CI, confidence interval.

**Table 3:** Multivariate Cox regression of significant prognostic factors.

Covariate	*p*	Exp(b)	95% CI of Exp(b)
Citrulline	0.010	0.919	0.86–0.98
Hemoglobin	0.009	0.679	0.51–0.91
PS	0.036	3.056	1.07–8.70

b, regression coefficient; Exp(b), relative risk; CI, confidence interval. Performance status (PS), ECOG performance status.

## Supplementary Materials

The following are available online at https://www.mdpi.com/2072-6694/12/7/1983/s1, Figure S1: Kaplan–Meier curves of overall survival in patients with metastatic soft tissue sarcoma, Figure S2: Correlation heat map of serum metabolites based on Pearson’s correlation coefficient, Figure S3: Quartile stratification of STS patients according to the risk score from the Cox regression model, Figure S4: Diagnostic discriminatory power of serum citrulline and hemoglobin, by receiving operator characteristic curve analysis, Table S1: Serum levels of 53 amino acids, 15 bile acids and urea, determined by metabolomics profiling in patients with metastatic soft tissue sarcoma.
