# Supplementary material for: Integration of Serum Metabolomics into Clinical Assessment to Improve Outcome Prediction of Metastatic Soft Tissue Sarcoma Patients Treated with Trabectedin
Source: Cancers (Basel). 2020 Jul 21;12(7):1983. doi: 10.3390/cancers12071983 (PMC7409362; doi:10.3390/cancers12071983)
Supplement: Supplementary file 1 [file cancers-12-01983-s001.pdf]

Supplementary material

Integration of Serum Metabolomics into Clinical Assessment to Improve Outcome Prediction of Metastatic Soft Tissue Sarcoma Patients Treated with Trabectedin

Gianmaria Miolo, Emanuela Di Gregorio, Asia Saorin, Davide Lombardi, Simona Scalone, Angela Buonadonna, Agostino Steffan and Giuseppe Corona

Table S1. Serum levels of 53 amino acids, 15 bile acids and urea, determined by metabolomics profiling in patients with metastatic soft tissue sarcoma.

Metabolite	Mean (SD), M	Median, M	Range
**Amino acid or derivative**			
3-Methylhistidine	3.98 (1.33)	4.01	1.81–7.59
2-Aminoadipic acid	NA	–	–
2-Aminobutyric acid	NA	–	–
3-Aminoisobutyric acid	NA	–	–
1-Methylhistidine	4.84 (5.72)	2.70	0.24–22.29
5-Hydroxylysine	0.20 (0.11)	0.18	0.10–0.62
Asymmetric dimethyl-arginine (ADMA)	0.53 (0.08)	0.52	0.33–0.71
Agmatine	6.88 (6.62)	5.02	3.18–35.09
Alanine	358.37 (114.16)	355.01	147.11–559.64
Anserine	NA	–	–
Arginine	59.37 (16.19)	59.22	34.46–97.09
Argininosuccinic acid	NA	–	–
Asparagine	44.71 (9.52)	44.11	22.45–63.56
Aspartic acid	7.39 (2.65)	6.78	3.43–14.77
Beta-alanine	NA	–	–
Carnosine	NA	–	–
Citrulline	33.64 (11.07)	34.79	12.22–58.95
Creatinine	85.35 (22.01)	86.34	42.85–120.70
Cystathionine	0.50 (0.29)	0.44	0.16–1.27
Cysteine	NA	–	–
Cystine	13.06 (13.11)	9.59	1.33–47.06
Ethanolamine	6.66 (1.47)	6.97	3.75–9.26
Gamma-aminobutyric acid	NA	–	–
Glutamic acid	95.24 (69.28)	78.65	30.46–376.71
Glutamine	585.21 (118.95)	595.57	199.51–764.53
Glycine	215.91 (62.36)	213.13	129.34–429.64
Histidine	73.41 (12.19)	76.46	46.67–92.08
Homocitrulline	2.48 (1.81)	2.25	0.83–8.81
Homocystine	NA	–	–
Isoleucine	59.04 (13.08)	59.59	35.71–86.48
Kynurenine	1.55 (0.62)	1.35	0.75–2.91
Leucine	146.39 (28.47)	151.36	80.66–187.99
Lysine	200.71 (35.44)	200.69	132.85–272.86
Methionine	27.06 (7.27)	28.60	14.83–45.21
Methionine sulfoxide	0.20 (0.11)	0.17	0.07–0.64
N-Acetylputrescine	NA	–	–
O-Phosphorylethanolamine	NA	–	–
O-Phosphoserine	NA	–	–

Ornithine	75.69 (16.92)	75.93	39.53–102.09
Phenylalanine	70.76 (11.40)	69.57	54.80–93.64
Phenylethylamine	0.48 (0.13)	0.46	0.13–0.71
Proline	234.76 (71.76)	256.47	91.43–339–48
Sarcosine	NA	–	–
Symmetric dimethyl-arginine (SDMA)	0.42 (0.09)	0.42	0.27–0.60
Serine	103.34 (21.61)	102.28	63.84–145.72
Serotonin	0.04 (0.06)	0.02	0.00–0.27
Taurine	12.26 (2.97)	11.53	6.90–19.23
Threonine	141.43 (30.29)	136.14	86.84–198.10
Trans-4-hydroxyproline	1.31 (0.69)	1.18	0.51–3.42
Tryptophan	46.13 (10.88)	46.12	16.17–62.91
Tyrosine	62.52 (15.45)	60.15	38.75–97.69
Urea	$6.4\ 10^3(1.6\ 10^3)$	5947.18	$4.0\ 10^3–1.1\ 10^3$
Valine	197.87 (29.68)	193.44	145.42–265.92
**Bile acid**			
Cholic acid (CA)	0.20 (0.31)	0.06	0.01–1.13
Chenodeoxycholic acid (CDCA)	0.29 (0.44)	0.18	0.011–1.87
Deoxycholic acid (DCA)	0.45 (0.44)	0.31	0.02–1.70
Glycocholic acid (GCA)	0.37 (0.39)	0.28	0.00–1.41
Glycochenodeoxycholic acid (GCDCA)	1.21 (0.94)	1.09	0.06–3.39
Glycodeoxycholic acid (GDCA)	0.49 (0.48)	0.32	0.01–1.72
Glycolithocholic acid (GLCA)	0.02 (0.03)	0.01	0.00–0.12
Glycoursodeoxycholic acid (GUDCA)	0.12 (0.19)	0.06	0.00–0.86
Lithocholic acid (LCA)	0.01 (0.01)	0.01	0.00–0.03
Taurocholic acid (TCA)	0.10 (0.18)	0.04	0.00–0.68
Taurochenodeoxycholic acid (TCDCA)	0.28 (0.34)	0.17	0.01–1.56
Taurodeoxycholic acid (TDCA)	0.09 (0.15)	0.06	0.01–0.74
Taurolithocholic acid (TLCA)	0.01 (0.004)	0.003	0.00–0.02
Tauroursodeoxycholic acid (TUDCA)	0.01 (0.01)	0.00	0.00–0.06
Ursodeoxycholic acid (UDCA)	0.07 (0.06)	0.04	0.01–0.24

NA, data not available, concentrations below the low limit of quantification.

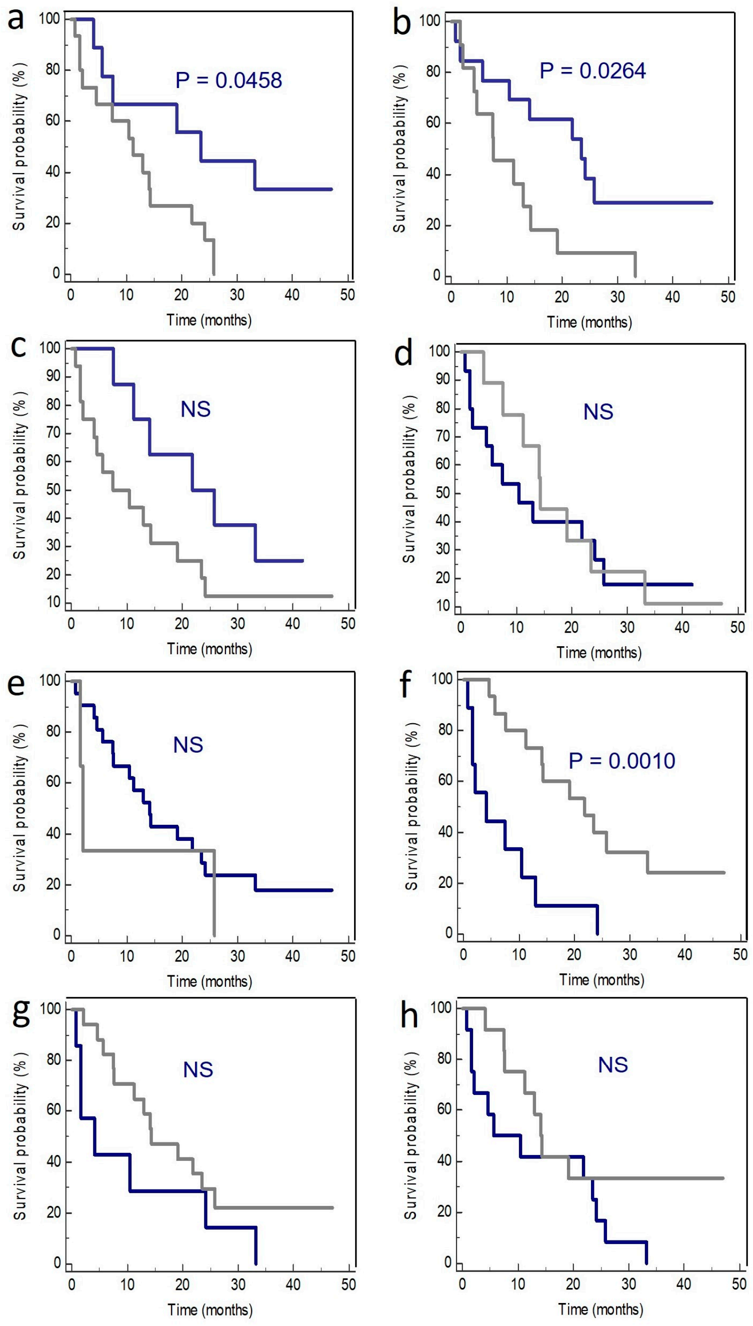

Figure S1. Kaplan-Meier curves of overall survival in patients with metastatic soft tissue sarcoma. **(a)** L-sarcomas (leiomyosarcomas and liposarcomas, n = 9, blue) vs. other histotypes (n = 15). **(b)** Performance status score of 0 (n = 13, blue) vs. 1 (n = 11). **(c)** Tumor grade G3 (n = 16, blue) vs. G2 (n = 8). **(d)** Age < 65 (n = 15, blue) vs. ≥ 65 years (n = 9). **(e)** Absolute neutrophil count < 7.5 10^9 cells/L (n = 21, blue) vs. ≥ 7.5·10^9/L (n = 3). **(f)** Hemoglobin < 12 g/dL (n = 9, blue) vs. ≥ 12 g/dL (n = 15). **(g)** Albumin < 3.5 g/dL (n = 7, blue) vs. ≥ 3.5 g/dL (n = 17). **(h)** Lactate dehydrogenase < 320 U/L (n = 12, blue) vs. ≥ 320 U/L (n = 12).

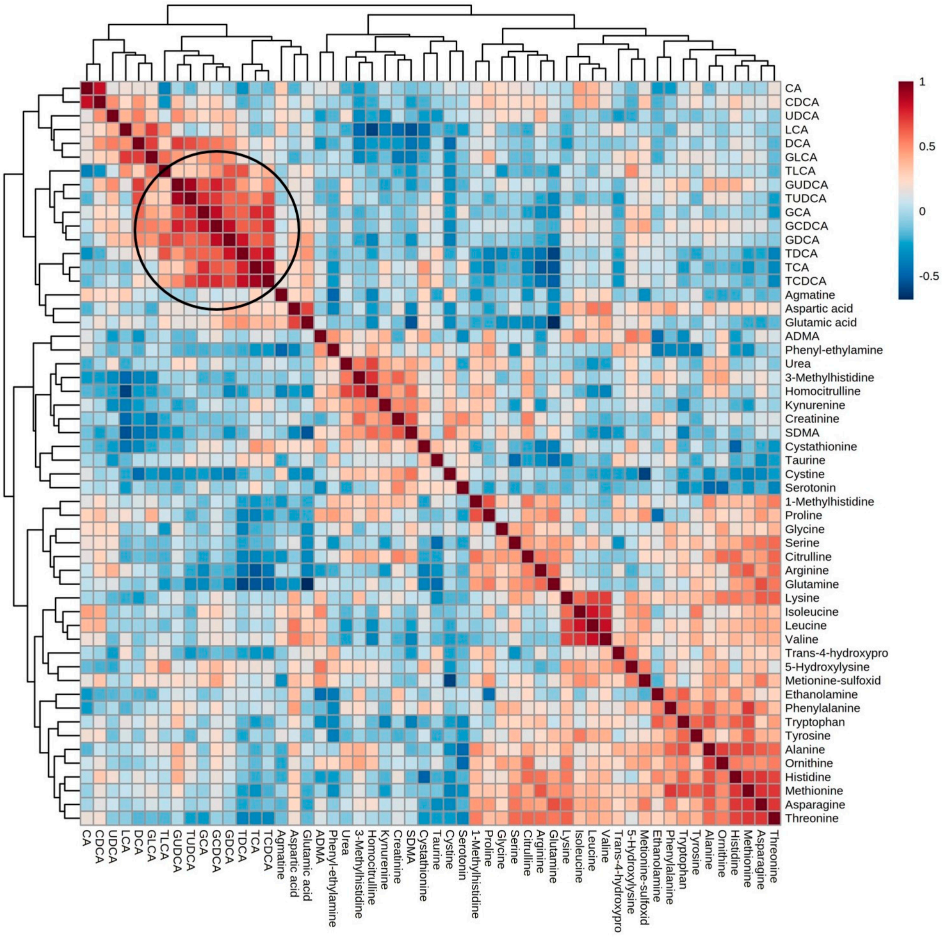

Figure S2. Correlation heat map of serum metabolites based on Pearson's correlation coefficient. Positive correlations are shown in red, and negative correlations are shown in blue. The circle marks a cluster of bile acids whose levels correlate. Abbreviations are defined in Table S1.

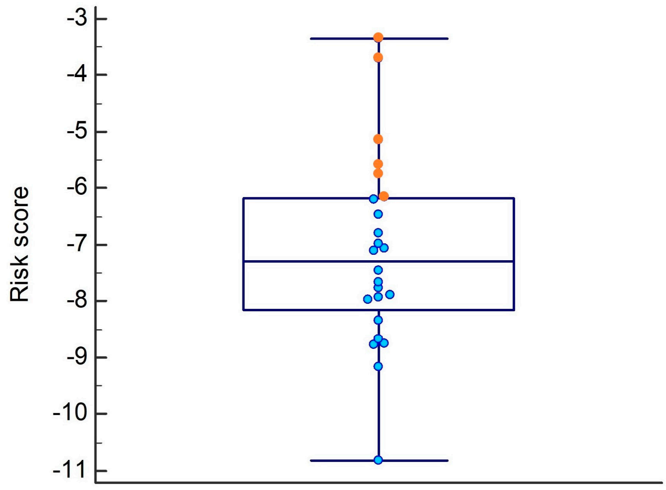

Figure S3. Quartile stratification of STS patients according to the risk score from the Cox regression model. In the box-and-whisker plot, the central box represents the values from the lower to upper quartile and the middle line the median. Patients with a risk score > or ≤ the upper quartile value (-6.19) were included in the H-Risk (orange) and LM-Risk groups (blue), respectively.

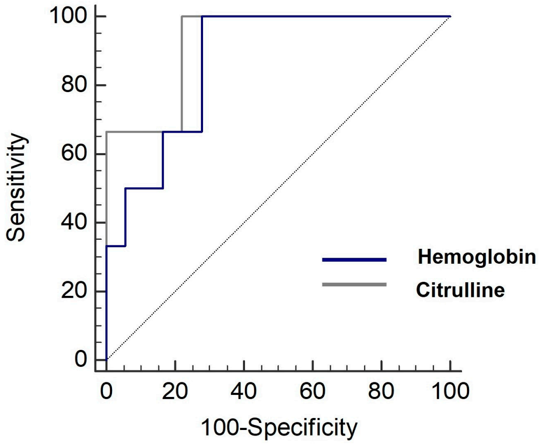

Figure S4. Diagnostic discriminatory power of serum citrulline and hemoglobin, by receiving operator characteristic curve analysis. For citrulline, the area under the curve was 0.926, with a sensitivity of 100% and a specificity of 77.8%. The cut-off value was 33.7 μM. For hemoglobin, the area under the curve was 0.870, with a sensitivity of 100% and a specificity of 72.2%. The cut-off value was of 12.4 g/dL.

© 2020 by the authors. Licensee MDPI, Basel, Switzerland. This article is an open access article distributed under the terms and conditions of the Creative Commons Attribution (CC BY) license (http://creativecommons.org/licenses/by/4.0/).
